# Early termination of pregnancy: differences in gestational age estimation using last menstrual period and ultrasound in Mexico

**DOI:** 10.1186/s12978-020-00914-x

**Published:** 2020-06-09

**Authors:** Biani Saavedra-Avendano, Raffaela Schiavon, Patricio Sanhueza, Ranulfo Rios-Polanco, Laura Garcia-Martinez, Blair G. Darney

**Affiliations:** 1 Centro de Investigación y Docencia Economicas (CIDE), Mexico City, Mexico; 2 Independent consultant, Mexico City, Mexico; 3 Secretaría de Salud de la Ciudad de México, Mexico City, Mexico; 4 Department of Obstetrics and Gynecology and School of Public Health Portland, Oregon Health & Science University, Mail code UHN-50, 3181 SW Sam Jackson Park Rd, Portland, OR 97239 USA; 5 Centro de Investigación en Salud Poblacional (CISP), Instituto Nacional de Salud Pública (INSP), Cuernvaca, Mexico

**Keywords:** Gestational age, Last menstrual period, Ultrasonography, Early abortion, Mexico, Edad gestacional, Fecha de última menstruación, Ultrasonido, Aborto temprano, México

## Abstract

**Background:**

Gestational age estimation is key to the provision of abortion, to ensure safety and successful termination of pregnancy. We compared gestational age based on reported last menstrual period and ultrasonography among a large sample of women in Mexico City’s public first trimester abortion program, *Interrupcion Legal de Embarazo (ILE)*.

**Methods:**

We conducted a retrospective study of 43,219 clinical records of women seeking abortion services in the public abortion program from 2007 to 2015. We extracted gestational age estimates in days based on last menstrual period and ultrasonography. We calculated the proportion of under- and over-estimation of gestational age based on last menstrual period versus ultrasonography. We compared overall differences in estimates and focused on discrepancies at two relevant cut-offs points (70 days for medication abortion eligibility and 90 days for *ILE* program eligibility).

**Results:**

On average, ultrasonography estimation was nearly 1 (− 0.97) days less than the last menstrual period estimation (SD = 13.9), indicating women tended to overestimate the duration of their pregnancy based on recall of date of last menstrual period. Overall, 51.4% of women overestimated and 38.5% underestimated their gestations based on last menstrual period. Using a 70-day limit, 93.8% of women who were eligible for medication abortion based on ultrasonography would have been correctly classified using last menstrual period estimation alone. Using the 90-day limit for ILE program eligibility, 96.0% would have been eligible for first trimester abortion based on last menstrual period estimation alone.

**Conclusions:**

The majority of women can estimate gestational age using last menstrual period date. Where available, ultrasonography can be used, but it should not be a barrier to providing care.

## Resumen

**Antecedentes:**

Estimar la edad gestacional durante el embarazo resulta esencial en provisión de servicios de aborto. Comparamos la edad gestacional con base en la fecha de última menstruación y de acuerdo a los resultados del ultrasonido, entre pacientes que solicitaron los servicios de aborto en el primer trimestre en el programa público de la Ciudad de México *Interrupción Legal del Embarazo.*

**Métodos:**

Revisamos de forma retrospectiva 43,219 expedientes clínicos de las mujeres que solicitaron servicios de aborto en el programa público de la Ciudad de México del periodo 2007 a 2015. Extrajimos la edad gestacional en días estimada mediante la fecha de última menstruación, así como por ultrasonido. Estimamos la proporción de subestimación y sobreestimación de la edad gestacional comparando el método que usa la fecha de última menstruación con los resultados del ultrasonido. Asimismo, usamos dos puntos de corte: 70 días (10 semanas de gestación) para aborto con medicamentos y 90 días (12 semanas y 6 días de gestación) como límite para la elegibilidad del programa, y estimamos la proporción de mujeres que rebasan dichos puntos de corte con base en la fecha de ultima menstruación.

**Resultados:**

En promedio, la estimación de la edad gestacional usando el ultrasonido fue de − 0.97 días menor comparado con le edad gestacional estimada mediante la fecha de última menstruación (DE = 13.9). Lo anterior indica que en general las mujeres sobreestiman la edad gestacional con base en la fecha de última menstruación. En total, 51.4% de las mujeres sobreestiman la edad gestacional y 38.5% la subestiman con base en la fecha de última menstruación. Usando el punto de corte de 70 días, 93.8% de las mujeres que fueron elegibles para recibir un aborto por medicamento con base en los resultados del ultrasonido, habrían sido correctamente seleccionadas usando solamente la fecha de última menstruación; 96.0% habrían sido elegibles para recibir los servicios de aborto en el primer trimestre (punto de corte de 90 días) usando solamente la fecha de última menstruación.

**Conclusiones:**

Las mujeres pueden estimar la edad gestacional usando la fecha de última menstruación. Cuando hay disponibilidad, el ultrasonido puede ser usado para estimar la edad gestacional. Sin embargo, la carencia de este instrumento de medición no debe ser una barrera para proveer servicios de aborto seguros y de calidad.

## Plain English summary

Gestational age estimation is key to the provision of abortion to ensure safe and successful termination of pregnancy. Requiring ultrasonography when such technology or trained professionals are not available may cause a delay in access to abortion and increase the cost of care. We compared gestational age based on self-reported last menstrual period and estimated using ultrasonography among a large sample of women in Mexico City’s public abortion program, *Interrupcion Legal de Embarazo* (ILE). In this program, medical abortion is used up to 70 days, and women are eligible to receive legal abortions up to 12 weeks 6 days (90 days). We used two cut-offs: 70 days for medical abortion and 90 days for ILE program eligibility.

Overall, 51.4% of women overestimated, and 38.5% underestimated gestational age based on last menstrual period compared with ultrasonography. The vast majority of women would have been correctly classified for medication abortion (93.8%) and ILE program eligibility (96.0%) using last menstrual period gestational age estimation alone. Women can correctly estimate gestational age using last menstrual period date. Requiring ultrasonography should not be a barrier to providing safe, legal abortion care.

## Background

Gestational age (GA) estimation is key to the provision of abortion, to ensure safe and successful termination of pregnancy [1]. Although international guidelines state that ultrasonography (US) can be used, if and when available, to measure GA prior to abortion care, [1] many still prefer to rely upon US to determine GA. However, evidence suggests that abortion can be safely provided in the absence of US technology [2–5] and that most women seeking first-trimester abortion services can calculate their pregnancy duration within a margin of error that would allow for safe abortion care [2–8]. Moreover, it has been shown that women who know their last menstrual period (LMP) date tend, on average, to overestimate pregnancy duration [5, 6, 8, 9].

In Mexico City, where first trimester abortion was decriminalized in 2007, medication and aspiration abortions are available to women on demand in public and private sectors up to 12 weeks and 6 days of gestation [10, 11]. Public-sector abortion services, known as the *Interrupcion Legal de Embarazo* (ILE) program, have provided over 210,000 first trimester abortions since services began in spring 2007 [12]. Medication abortion is offered up to 10 weeks (70 days); aspiration abortion after 10 weeks and up to the 12 week and 6 days legal limit (90 days), or to women who reside outside Mexico City in order to provide a completed abortion in a single day.

The Mexico City Ministry of Health guidelines [10] explicitly state that US is prioritized over LMP to determine gestational age and thus ILE program eligibility. Second trimester abortion is highly restricted and available only under narrow indications (e.g. rape, fetal anomalies, threat to health or life of the woman) [13, 14]. However, omitting the routine requirement for US prior to an abortion in Mexico has the potential to improve overall access to timely abortion care and reduce delays and costs without impacting the safe provision of services.

We aimed to quantify discrepancies in GA by LMP and US dating, and to estimate eligibility for medication (70 days) and all first trimester (90 days) abortion using LMP alone. Based on previous literature, we hypothesized that a high proportion of women would be able to correctly estimate the duration of their pregnancy using LMP, and that they would more commonly overestimate GA using LMP compared with US.

## Methods

We conducted a retrospective chart review study using a sample of clinical data from three outpatient sites in the Mexico City ILE program, from 2007 to 2015 (*N* = 48,241). Details of data abstraction and quality checking are reported elsewhere [15]. We used data from all women who sought abortion services and had GA information, regardless of whether they received the procedure or not (due to suspected ectopic pregnancy, referral to other institution, or presenting past the GA limit for legal abortion in Mexico City) [16]. We excluded *n* = 5022 observations due to missing or implausible gestational age data (e.g. 40 weeks) based on LMP, US, or both (see Additional file 1; Table S1 for differences between included and excluded observations).

Our outcomes are two measurements of GA: calculated by the physician based on the woman’s reported date of LMP, and estimated by US. We created a variable to measure the absolute difference in days between GA estimated by US and by LMP. Based on the ILE program’s clinical guidelines (women <= 70 days’ GA routinely receive medication abortion) and Mexico City’s abortion law (only first trimester procedures are legal on request), we established two GA cut-offs at 70 days (10 weeks) and 90 days (12 weeks and 6 days) of gestation.

We included several socio-demographic variables: age, marital status, the woman’s highest completed grade or level of schooling, occupation, number of pregnancies (including the current pregnancy), and state of residence. We also included the type of abortion procedure (aspiration or medication), with separate categories for women who did not receive a procedure due to presenting past the GA limit, or due to suspected ectopic pregnancy or referral to another institution (“other” category).

We described women’s socio-demographic characteristics and GA based on LMP and US. We used visualizations to describe GA distribution based on LMP and US (histogram) and the relationship between the two estimation methods (LMP and US; scatterplot and correlation coefficient). We next calculated the proportion of over- and under-estimation of GA based on LMP, compared with US, overall and using cut-offs at 70 and 90 days. Finally, we tested for differences in socio-demographic characteristics between women who over- or under-estimated their GA by LMP compared with US by fewer than 8 days and greater than 7 days.

We also examined outliers in our data (although we excluded implausible GA values [Table S1], we included all possible values, including outliers). We assessed two different types of outliers: in GA estimation based on US and LMP (Table S2) and outliers in the discrepancy between the two estimations (Table S3). We replicated our primary analysis (depicted in Table 2), excluding 535 observations (1.2% of the total sample) over 15 weeks of gestation estimated by US or LMP (Table S4). We also replicated the analysis removing 469 outliers (1.1%) at the extremes of the discrepancy in GA between US and LMP distribution (Table S5). Our results were robust to these sensitivity analyses. We present our main results with all observations. The Research Ethics Committee at the National Institute of Public Health, Cuernavaca (1746), the Research and Teaching Committee at the Secretaria de Salud, Mexico City (101–110–12-15), and the Oregon Health & Science University IRB approved this study. We used stata 14 for all analyses.

## Results

Our final analytical sample included 43,219 observations. Table 1 presents the women’s socio-demographic characteristics. Most women were between 18 and 24 (48.0%) and 25–29 years (21.2%). Half the sample (49.8%) was married or cohabitating, 46.9% reported any type of employment outside the home, 27.2% were students, and 70.9% resided in Mexico City. Over one third of the women (36.8%) had not experienced a pregnancy previous to the one they were seeking to terminate. Overall, 69.7% received a medication abortion, 19.5% an aspiration, while the remainer did not receive an abortion either because they presented past the gestational limit (7.4%) or for another reason (3.4%).

**Table 1:** Socio-demographic characteristics of women who sought abortion services in the Mexico City public program

Socio- demographic characteristics	Analytical Sample ***N*** = 43,219 (100%)	
	n	%
**Age (years)**		
12-17^a^	3710	8.6
18–24	20,735	48.0
25–29	9161	21.2
30–39	8439	19.5
40-max	1101	2.6
*missing*	73	0.2
**Marital Status**		
Never Married	17,806	41.2
Married /Cohabitating	21,526	49.8
Widowed/ Divorced	2356	5.5
*missing*	1531	3.5
**Educational level**		
Primary	3695	8.6
Secondary	13,858	32.1
High School	16,667	38.6
University	7384	17.1
*missing*	1615	3.7
**Employment**		
Unemployed	9572	22.2
Employed	20,256	46.9
Students	11,758	27.2
*missing*	1633	3.8
**Gravidity**^**b**^		
One	15,908	36.8
2 or 3	18,991	43.9
4 or greater	7424	17.2
*missing*	896	2.1
**Place of residence**		
Mexico City	30,661	70.9
State of Mexico	10,219	23.6
Other State	2289	5.3
*missing*	50	0.1
**Procedure**		
Medication abortion	30,123	69.7
Aspiration abortion	8436	19.5
Did not receive abortion	3182	7.4
Other^c^	1478	3.4

Note: ^a^ Women under 18 are required to have permission from a parent or guardian permission to access an abortion in the ILE program. ^b^ Including the current pregnancy. ^c^ “Other” category includes: suspected ectopic pregnancy or referral to another institution

**Fig. 1:**
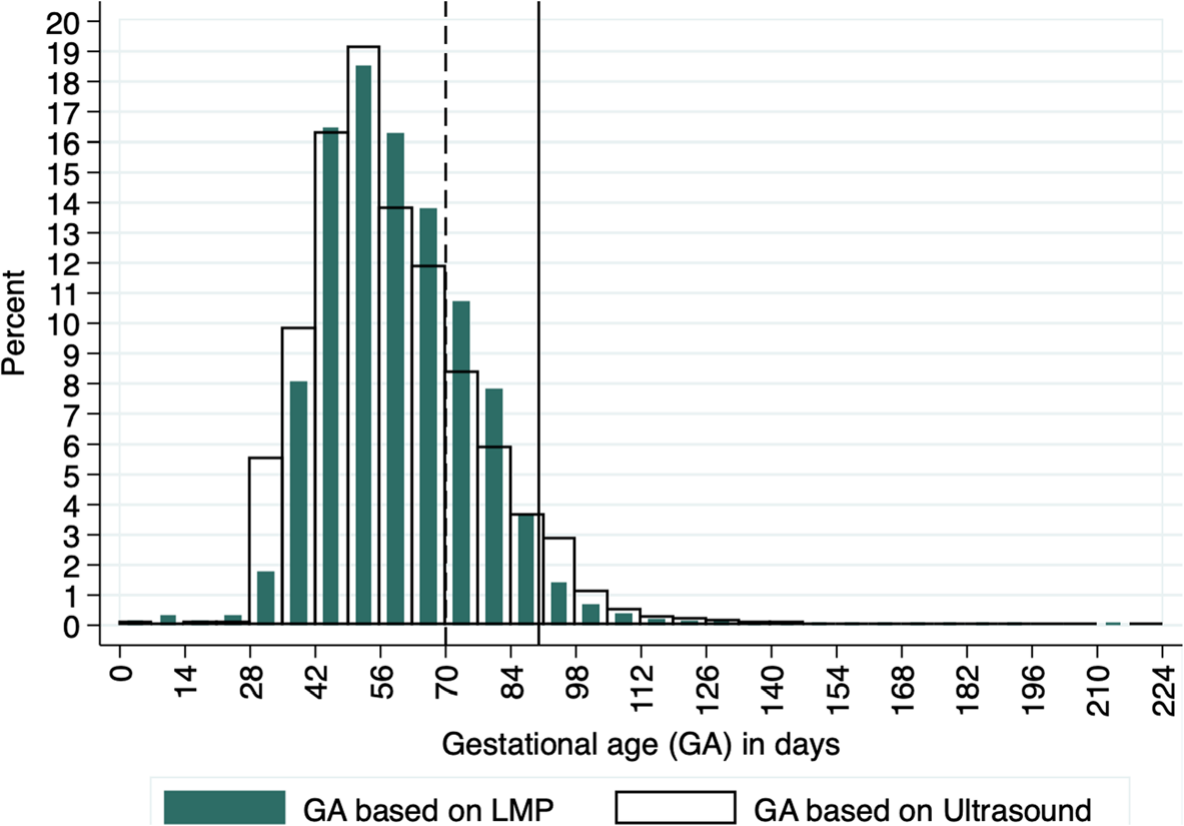
GA distribution (days) estimated based on LMP and US with cut-offs at 70 and 90 days, overall sample *N* = 43,219

Figure 1 depicts GA distributions by LMP and by US; the LMP curve is slightly displaced to the right, visually showing overestimation of GA by LMP compared with US between 56 and 84 days. Mean GA was 58.1 days based on US (SD = 18.1; range = 0–220) and 59.1 days based on LMP (SD = 15.8; range = 0–216); the mean difference between the two estimations was − 0.97 day (SD = 13.9; range = − 130 – 165).

**Fig. 2:**
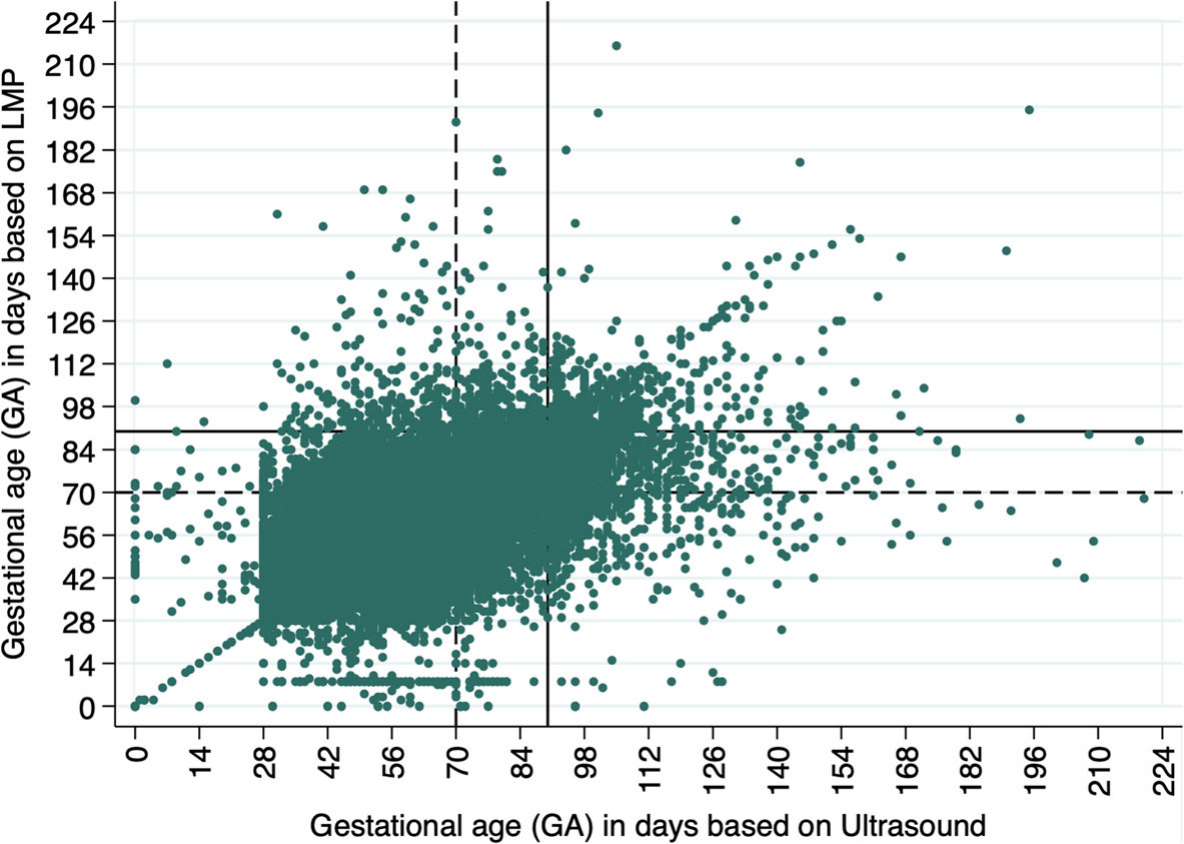
Relationship between GA estimated based on LMP and US with 70 and 90 day cutoffs, overall sample N = 43,219

Figure 2 shows the relationship between GA estimation by LMP and US. The two measures of GA were highly correlated (r = 0.7; *p* < 0.001) [17]. Overall, 51.4% of women in our sample (22,201/43,219; Table 2, line 2) overestimated their GA based on LMP compared to US measurement, while 38.5% of them (16,637/ 43,219; Table 2, line 3) underestimated their GA compared with US. One out of 10 women in the sample had zero difference between GA estimated by LMP and US (4381/43,219; Table 2, line 1). The discrepancy between the two estimations was fewer than 5 days (+/− 4 days) in 43.9% of the sample and fewer than 8 days (+/− 7 days) in 61.6% of the sample (Table 2, line 1.a & 1.b).

**Table 2:** Proportion of under/over-estimation of GA based on LMP compared with US, *N* = 43,219, Mexico City ILE program

	Overall Sample		
		n	%
	Total	43,219	100
**1**	**No differences in GA between LMP & US**	**4381**	**10.1**
1.a	Discrepancy between LMP & US +/− 4 days	19,016	43.9
1.b	Discrepancy between LMP & US +/− 7 days	26,644	61.6
**2**	**Over-estimation of GA by LMP**	**22,201**	**51.4**
2.a	GA > 70 days based on LMP & < =70 based on US	3080	7.1
2.b	GA > 90 days based on LMP & < =90 based on US	531	1.2
**3**	**Under-estimation of GA by LMP**	**16,637**	**38.5**
3.a	GA < =70 days based on LMP & > 70 based on US	2701	6.2
3.b	GA < =90 days based on LMP & > 90 based on US	1709	4.0

Note: Percentages in bold add 100%

We calculate that 6.2% of women (2701/43,219; Table 2, line 3.a) would have been offered medical abortion based on LMP despite being ineligible per US (70 day cut-off). According on the 12 week and 6 day cut-off (90 days), 4.0% (1709/43,219; Table 2, line 3.b) of women would have received legal first trimester abortion based on LMP despite being ineligible by US measurement. That is, for 96% of women, US measurement did not alter their eligibility for a legal abortion in the ILE program.

Based on LMP alone, 7.1%, (3080/43,219; Table 2, line 2.a) of the sample would have been ineligible for medication abortion despite being eligible based on US measurement; and 1.2% (531/43,219; Table 2, line 2.b) would have been ineligible for first trimester abortion services based on LMP, but being eligible based on US.

Figure S1 (see Additional file 2) focuses on the subsample of women who underestimated GA at the 70 day cut-off (*n* = 2701; Table 2, line 3.a). Of those, 23.2% (626/2701; 1.4% of the total sample) had differences in GA between LMP and US by 1 to 7 days, 20.3% (548/2701; 1.2% of the full sample) had differences in GA by 8 to 14 days and 30.5% (825/2701; 1.9% of the total sample) of the subsample subestimated their GA by more than 28 days.

We found few meaningful sociodemographic differences between women who over- or under-estimated their GA from 0 to 7 days compared with those whose GA estimate by LMP differed by more than 7 days. A larger proportion of women with discrepancies in GA greater than 7 days between LMP and US did not receive an abortion due to being past the GA limit (13.4% vs. 3.6%; Additional file 1; Table S6).

## Discussion

In the Mexico City public abortion program, ILE, women’s GA based on reported LMP was, on average, very close to the GA based on US but slight overestimated GA, with a mean difference − 0.97 days. We confirmed that women mostly tend to overestimate their duration of pregnancy using LMP: 51.4% of the women in our sample overestimated, while 38.5% underestimated it. Our results show that, using LMP alone, 93.8% of women who received medication abortion (70 day limit) would have been correctly classified as eligibile and 96.0% would have correctly received legal first trimester abortion (90 day limit). US measurement, therefore, did not alter either clinical eligibility for medication abortion nor legal eligibility for abortion services, compared to using only LMP.

Previous studies have reported a variable proportion (0.9–12%) of women underestimating their GA by LMP compared with US, thus falling outside the limit for medication abortion (using a 63 day limit) [6, 9, 17, 18]. We found that while over a third of our sample underestimated GA using LMP compared with US, only 6.2% of our sample underestimated using LMP such that they were not eligible for medication abortion per US, in the middle of previously described range. The clinical and programmatic implications of such results, however, must be interpreted with caution, since a safe and successful termination of pregnancy by medication abortion has been well documented beyond 70 days (10 weeks) [1, 19]. In addition, where US is not used prior to medication abortion, studies have reported low complication rates, similar to when US is used [4, 5, 20, 21].

The mean difference between LMP and US in GA estimations that we found is strikingly similar to findings of a study among women who continued their pregnancies, which estimated GA at birth through first trimester report of LMP and US (mean difference: 0.8 days) [22]. The discrepancy between the two estimations in our study was within the documented uncertainty range of US itself (+/− 4 days) [23] in 44% of the sample, and did not exceed 1 week in six out of ten women studied.

Our results show, on the other end, that LMP-only based overestimation of GA would have prevented 7.1% of women from accessing medication abortion and 1.2% from accessing legal abortion altogether, if an US would not have been performed. Overall, it is reasonable to state that women’s safety in our study would have not been compromised by using GA based on LMP only. However, legal eligibility could have, in a small proportion (4%) of cases.

While early abortion can be delivered effectively and safely to most women without the use of US, [1] there will be women who could benefit from its use. Women with difficulty recalling LMP, with irregular menstrual cycles, or with known or suspected risk factors such as uterine fibroids, uterine anomalies, twin pregnancy, or ectopic pregnancy.

This study has limitations. First, our calculations assume that GA estimated by US is always accurate. However, US is known to have a +/− 4 days margin of error, and thus can also have resulted in slightly inaccurate GA estimates [23]. Second, although we standardized chart abstraction, it is possible that clinical processes were not entirely standardized across facilities; we do not know the exact timing of the report of LMP during the course of the clinical visit. Third, we used physicians’ estimation of GA based on LMP reported by women, instead of our estimations using LMP and abortion dates. Fourth, there was no supporting information in the clinical history to determine how confident women were of the LMP they reported, or how sure they were in their recall. Fifth, we were unable to access additional clinical information that could be useful in this context to increase diagnostic sensitivity and to rule out later gestations such as strategies to improve women’s recall, posing specific questions to identify signs and symptoms of later pregnancy, or performing abdominal palpation or bimanual pelvic exam [1, 24, 25]. Sixth, we excluded from our analytical sample women with missing values or implausible data in GA estimation. A greater proportion of excluded women (21.7%) did not receive abortion care due to presenting past the gestational age limit, these women tend to have incomplete medical charts overall.

Finally, while we analyzed a large sample of women who sought abortion services in the ILE program, it does not represent all abortions performed in the public program. However, when we compared this sample with the overall aggregate profile of all ILE users [12], we found that they are similar, except for a higher proportion of adolescents (8.6% in the sample versus 6.4% in overall ILE program), due to the inclusion of a referral facility for adolescents in our study.

## Conclusions

This study provides robust information about GA estimation in a large sample of women seeking legal abortion services in a Latin American population. Our results confirm women’s ability to assess GA using LMP; the women in our sample could establish their eligibility for medication abortion protocols and for legal first trimester abortion eligibility overall. Requiring US for GA dating when the technology is not accessible may cause a delay in care, which in itself is a barrier to accessing early abortion and a risk factor for complications [26]. Where available, US can be used, but consistent with global guidelines should not be a barrier to providing care [1]. Our findings support existing international guidelines [1, 24, 27] and have direct implications for regulations, norms, and protocols for the provision of abortion services in Mexico and the Latin American region, especially in settings with scare resources.

## Supplementary information

**Additional file 1: Table S1.** Socio-demographic characteristics of the analytical sample and excluded women because of missing or implausible GA data. **Table S2.** GA distribution estimated based on US and LMP: outliers’ analysis. **Table S3.** The Discrepancy in GA between US and LMP distribution: outliers’ analysis. **Table S4.** Proportion of over/under-estimation of GA based on LMP compared with US, sample excluding GA estimation outliers (GA > =112 days) *n* = 42,668. **Table S5.** Proportion of over/under-estimation of GA based on LMP compared with US, sample excluding outliers in the discrepancy distribution n = 42,750. **Table S6.** Socio-demographic characteristics among women who over- or under-estimated their GA by LMP more than 8 days compared with US and those women who had discrepancies of 0–7 days.

**Additional file 2: Figure S1.** Relationship between GA based on LMP and by US, among women who under-estimated of GA by LMP (<=70 days), *n* = 2701 (6.2% of total sample)

## Data Availability

The data that support the findings of this study are available from the Mexico City’s Ministry of Health but restrictions apply to the availability of these data, which were used under license for the current study, and so are not publicly available. Data are however available from the authors upon reasonable request and with permission of the Mexico City’s Ministry of Health.

## Abbreviations

GA: Gestational age
LMP: Last menstrual period
US: Ultrasonography
ILE: *Interrupcion legal del embarazo*
