# Supplementary material for: Early termination of pregnancy: differences in gestational age estimation using last menstrual period and ultrasound in Mexico
Source: Reprod Health. 2020 Jun 9;17:89. doi: 10.1186/s12978-020-00914-x (PMC7285429; doi:10.1186/s12978-020-00914-x)
Supplement: Supplementary file 1 — Additional file 1: Table S1. Socio-demographic characteristics of the analytical sample and excluded women because of missing or implausible GA data. Table S2. GA distribution estimated based on US and LMP: outliers’ analysis. Table S3. The Discrepancy in GA between US and LMP distribution: outliers’ analysis. Table S4. Proportion of over/under-estimation of GA based on LMP compared with US, sample excluding GA estimation outliers (GA > =112 days) n = 42,668. Table S5. Proportion of over/under-estimation of GA based on LMP compared with US, sample excluding outliers in the discrepancy distribution n = 42,750. Table S6. Socio-demographic characteristics among women who over- or under-estimated their GA by LMP more than 8 days compared with US and those women who had discrepancies of 0–7 days. [file 12978_2020_914_MOESM1_ESM.docx]

**Additional file 1.**

**Table S1. Socio-demographic characteristics of the analytical sample and excluded women because of missing or implausible GA data**

|  | **Analytical Sample** | | **Excluded Charts** | |
| --- | --- | --- | --- | --- |
|  | n | % | n | % |
|  | 43,219 | 89.6 | 5,022 | 10.4 |
| **Age (years)**** |  |  |  |  |
| 12-17^a^ | 3,710 | 8.6 | 556 | 11.1 |
| 18-24 | 20,735 | 48.0 | 2,406 | 47.9 |
| 25-29 | 9,161 | 21.2 | 1,005 | 20.0 |
| 30-39 | 8,439 | 19.5 | 863 | 17.2 |
| 40-max | 1,101 | 2.6 | 135 | 2.7 |
| *missing* | 73 | 0.2 | 57 | 1.1 |
| **Marital Status**** |  |  |  |  |
| Single | 17,806 | 41.2 | 1,500 | 29.9 |
| Married /Cohabitating | 21,526 | 49.8 | 2,660 | 53.0 |
| Widowed/ Divorced | 2,356 | 5.5 | 235 | 4.7 |
| *missing* | 1,531 | 3.5 | 627 | 12.5 |
| **Educational level **** |  |  |  |  |
| Primary | 3,695 | 8.6 | 475 | 9.5 |
| Secondary | 13,858 | 32.1 | 1,568 | 31.2 |
| High School | 16,667 | 38.6 | 1,709 | 34.0 |
| University | 7,384 | 17.1 | 620 | 12.4 |
| *missing* | 1,615 | 3.7 | 650 | 12.9 |
| **Employment**** |  |  |  |  |
| Unemployed | 9,572 | 22.2 | 793 | 15.8 |
| Employed | 20,256 | 46.9 | 2,353 | 46.9 |
| Student | 11,758 | 27.2 | 1,213 | 24.2 |
| *missing* | 1,633 | 3.8 | 663 | 13.2 |
| **Gravidity^b^**** |  |  |  |  |
| One | 15,908 | 36.8 | 1,487 | 29.6 |
| 2 or 3 | 18,991 | 43.9 | 1,836 | 36.6 |
| 4 or greater | 7,424 | 17.2 | 720 | 14.3 |
| *missing* | 896 | 2.1 | 979 | 19.5 |
| **Place of residence** |  |  |  |  |
| Mexico City | 30,661 | 70.9 | 3,539 | 70.5 |
| State of Mexico | 10,219 | 23.6 | 1,179 | 23.5 |
| Other State | 2,289 | 5.3 | 260 | 5.2 |
| *missing* | 50 | 0.1 | 44 | 0.9 |
| **Procedure**** |  |  |  |  |
| Medication abortion | 30,123 | 69.7 | 2,273 | 45.3 |
| Aspiration abortion | 8,436 | 19.5 | 891 | 17.7 |
| Did not receive abortion | 3,182 | 7.4 | 1,088 | 21.7 |
| Other ^c^ | 1,070 | 2.5 | 525 | 10.5 |
| *missing* | 408 | 0.9 | 245 | 4.9 |

Note: ** p<0.001 Chi square test ^a^ Women under 18 are required to have permission from a parent or guardian permission to access an abortion in the ILE program. ^b^ Including the current pregnancy. ^c^ “Other” category includes: suspected ectopic pregnancy or referral to another institution.

**Table S2. GA distribution estimated based on US and LMP: outliers’ analysis**

| **GA in weeks** | **GA range in days** | **GA by US** | | | **GA by FUM** | | |
| --- | --- | --- | --- | --- | --- | --- | --- |
|  |  | n | % | Cum% | n | % | Cum% |
| **0 to10** | **0 to 70 days** | 33,819 | 78.25 | 78.25 | 33,439 | 77.37 | 77.37 |
| **11 to12** | **71 to 90 days** | 7,045 | 16.3 | 94.55 | 8,601 | 19.9 | 97.27 |
| **13** | **91 to 97 days** | 1,232 | 2.85 | 97.40 | 527 | 1.22 | 98.49 |
| **14** | **98 to 104 days** | 488 | 1.13 | 98.53 | 328 | 0.76 | 99.25 |
| **15** | **105 to 111 days** | 216 | 0.50 | 99.03 | 151 | 0.35 | 99.60 |
| **16** | **112 to 118 days** | 125 | 0.29 | 99.32 | 56 | 0.13 | 99.73 |
| **17** | **119 to 125 days** | 91 | 0.21 | 99.53 | 30 | 0.07 | 99.80 |
| **18** | **126 to 132 days** | 69 | 0.16 | 99.69 | 26 | 0.06 | 99.86 |
| **19** | **133 to 139 days** | 39 | 0.09 | 99.78 | 13 | 0.03 | 99.89 |
| **>=20** | **140 to 220 days** | 95 | 0.22 | 100.00 | 48 | 0.11 | 100.00 |
|  | **Total** | 43,219 | 100.00 |  | 43,219 | 100.00 |  |

Note: Outliers marked in black box (n=535; 1.2% of the total sample).

**Table S3. The Discrepancy in GA between US and LMP distribution: outliers’ analysis**

| **Discrepancy in GA (in weeks)**  **Between US and LMP dating** | **Discrepancy in GA (in days)**  **Between US and LMP dating** | **n** | **%** | **Cum %** |
| --- | --- | --- | --- | --- |
|  |  |  |  |  |
| **<=-9** | **-64 to -130** | 68 | 0.16 | 0.16 |
| **-7 to -8** | **-63 to -50** | 86 | 0.20 | 0.36 |
| **-5 to -6** | **-49 to -36** | 367 | 0.85 | 1.21 |
| **-4** | **-35 to -22** | 1495 | 3.46 | 4.67 |
| **-3** | **-21 to -15** | 2243 | 5.19 | 9.86 |
| **-2** | **-14 to - 8** | 5602 | 12.96 | 22.82 |
| **-1** | **-7 to -5** | 4508 | 10.43 | 33.25 |
|  | **-4 to -1** | 7831 | 18.12 | 51.37 |
| **Zero** | **0** | 4382 | 10.14 | 61.51 |
|  | **1 to 4** | 6803 | 15.74 | 77.25 |
| **1** | **5 to 7** | 3120 | 7.22 | 84.47 |
| **2** | **8 to 14** | 3419 | 7.91 | 92.38 |
| **3** | **15 to 21** | 1211 | 2.80 | 95.18 |
| **4** | **22 to 35** | 1292 | 2.99 | 98.17 |
| **5 to 6** | **36 to 49** | 475 | 1.10 | 99.27 |
| **7 to 8** | **50 to 63** | 164 | 0.38 | 99.65 |
| **9 to 18** | **64 to 126** | 142 | 0.33 | 99.98 |
| **>=19** | **127 to 165** | 9 | 0.02 | 100.00 |
|  | **Total** | 43,219 | 100.0 |  |

Note: Outliers marked in black box (n=468; 1.1% of the total sample).

**Table S4. Proportion of over/under-estimation of gestational age based on LMP compared with US, sample excluding GA estimation outliers (GA>=112 days) n=42,668**

|  |  | **n** | **%** |
| --- | --- | --- | --- |
|  | Total | 42,668 | 100 |
|  |  |  |  |
| **1** | **No differences in GA between LMP & US** | **4,366** | **10.2** |
| **2** | **Over-estimation of GA by LMP** | **22,072** | **51.7** |
| 2.a | GA >70 days based on LMP & <=70 based on US | 3,034 | **7.1** |
| 2.b | GA >90 days based on LMP & <=90 based on US | 448 | **1.0** |
| **3** | **Under-estimation of GA by LMP** | **16,246** | **38.1** |
| 3.a | GA <=70 days based on LMP & >70 based on US | 2,584 | **6.1** |
| 3.b | GA <=90 days based on LMP & >90 based on US | 1,429 | **3.3** |

Note: The mean discrepancy in GA between US and LMP estimation is -1.28 days (SD=12.37; range= -100 – 111).

**Table S5. Proportion of over/under-estimation of gestational age based on LMP compared with US, sample excluding outliers in the discrepancy distribution**

**n= 42,750**

|  |  | **n** | **%** |
| --- | --- | --- | --- |
|  | Total | 42,750 | 100 |
|  |  |  |  |
| **1** | **No differences in GA between LMP & US** | **4,381** | **10.2** |
| **2** | **Over-estimation of GA by LMP** | **22,047** | **51.6** |
| 2.a | GA >70 days based on LMP & <=70 based on US | 2,954 | 6.9 |
| 2.b | GA >90 days based on LMP & <=90 based on US | 426 | 1.0 |
| **3** | **Under-estimation of GA by LMP** | **16,322** | **38.2** |
| 3.a | GA <=70 days based on LMP & >70 based on US | 2,490 | 5.8 |
| 3.b | GA <=90 days based on LMP & >90 based on US | 1,493 | 3.5 |

Note: the mean discrepancy in GA between US and LMP estimation is -1.24 days (SD=11.79; range= -49 – 49).

**Table S6. Socio-demographic characteristics among women who over- or under-estimated their GA by LMP more than 8 days compared with US and those women who had discrepancies of 0-7 days**

|  | **Difference in GA between LMP and US: 0 to 7 days** | | **Difference in GA between LMP and US: >7 days** | |
| --- | --- | --- | --- | --- |
|  | n | % | n | % |
|  | 26,643 | 61.6 | 16,576 | 38.3 |
| **Age (years)**** |  |  |  |  |
| 12-17^a^ | 2,160 | 8.1 | 1,550 | 9.4 |
| 18-24 | 12,772 | 47.9 | 7,963 | 48.0 |
| 25-29 | 5,720 | 21.5 | 3,441 | 20.8 |
| 30-39 | 5,296 | 19.9 | 3,143 | 19.0 |
| 40-max | 649 | 2.4 | 452 | 2.7 |
| *missing* | 46 | 0.2 | 27 | 0.2 |
| **Marital Status** |  |  |  |  |
| Single | 11,009 | 41.3 | 6,797 | 41.0 |
| Married /Cohabitating | 13,295 | 49.9 | 8,231 | 49.7 |
| Widowed/ Divorced | 1,424 | 5.3 | 932 | 5.6 |
| *missing* | 915 | 3.4 | 616 | 3.7 |
| **Educational level **** |  |  |  |  |
| Primary | 2,198 | 8.3 | 1,497 | 9.0 |
| Secundary | 8,426 | 31.6 | 5,432 | 32.8 |
| High School | 10,293 | 38.6 | 6,374 | 38.5 |
| University | 4,762 | 17.9 | 2,622 | 15.8 |
| *missing* | 964 | 3.6 | 651 | 3.9 |
| **Employment*** |  |  |  |  |
| Unemployed | 5,816 | 21.8 | 3,756 | 22.7 |
| Employed | 12,624 | 47.4 | 7,632 | 46.0 |
| Students | 7,232 | 27.1 | 4,526 | 27.3 |
| *missing* | 971 | 3.6 | 662 | 4.0 |
| **Gravidity^b^**** |  |  |  |  |
| One | 9,871 | 37.1 | 6,037 | 36.4 |
| 2 or 3 | 11,670 | 43.8 | 7,321 | 44.2 |
| 4 or greater | 4,597 | 17.3 | 2,827 | 17.1 |
| *missing* | 505 | 1.9 | 391 | 2.4 |
| **Place of residence** |  |  |  |  |
| Mexico City | 18,932 | 71.1 | 11,729 | 70.8 |
| State of Mexico | 6,231 | 23.4 | 3,988 | 24.1 |
| Other State | 1,445 | 5.4 | 844 | 5.1 |
| *missing* | 35 | 0.1 | 15 | 0.1 |
| **Procedure**** |  |  |  |  |
| Medication abortion | 18,908 | 71.0 | 11,215 | 67.7 |
| Aspiration abortion | 5,398 | 20.3 | 3,038 | 18.3 |
| Did not receive abortion | 967 | 3.6 | 2,215 | 13.4 |
| Other ^c^ | 1,032 | 3.9 | 38 | 0.2 |
| *missing* | 338 | 1.3 | 70 | 0.4 |

Note: ** p<0.001 Chi square test ^a^ Women under 18 are required to have permission from a parent or guardian permission to access an abortion in the ILE program. ^b^ Including the current pregnancy. ^c^ “Other” category includes: suspected ectopic pregnancy or referral to another institution.
