# Supplementary figures and images for: Early termination of pregnancy: differences in gestational age estimation using last menstrual period and ultrasound in Mexico
Source: Reprod Health. 2020 Jun 9;17:89. doi: 10.1186/s12978-020-00914-x (PMC7285429; doi:10.1186/s12978-020-00914-x)

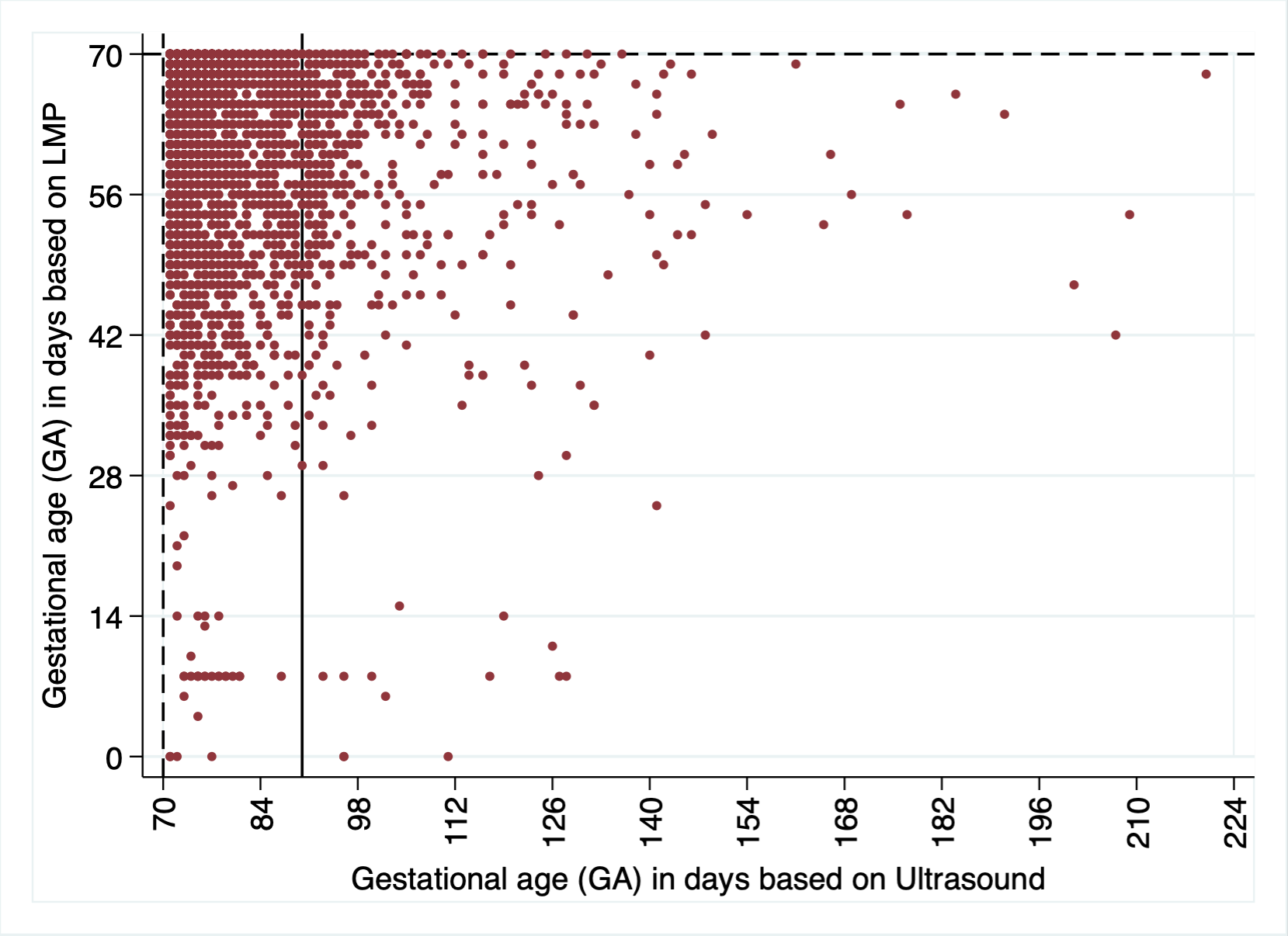

Supplement: Supplementary file 2 — Additional file 2: Figure S1. Relationship between GA based on LMP and by US, among women who under-estimated of GA by LMP (<=70 days), n = 2701 (6.2% of total sample) [file 12978_2020_914_MOESM2_ESM.tif]
